# Autoantibodies Against Ubiquitous and Confined Antigens in Patients With Ocular, Neuro-Ophthalmic and Congenital Cerebral Toxoplasmosis

**DOI:** 10.3389/fimmu.2021.606963

**Published:** 2021-05-12

**Authors:** Monica Goldberg-Murow, Carlos Cedillo-Peláez, Luz Elena Concha-del-Río, Rashel Cheja-Kalb, María José Salgar-Henao, Eduardo Orozco-Velasco, Héctor Luna-Pastén, Fernando Gómez-Chávez, Antonio Ibarra, Dolores Correa

**Affiliations:** ^1^ Laboratorio de Inmunología Exprimental, Instituto Nacional de Pediatría, CDMX, Mexico; ^2^ Centro de Investigación de Ciencias de la Salud, Universidad Anáhuac, Huixquilucan, Mexico; ^3^ Clínica de Enfermedades Inflamatorias Oculares, Asociación Para Evitar la Ceguera en México, I.A.P., CDMX, Mexico; ^4^ Cátedras CONACyT-Instituto Nacional de Pediatría, CDMX, Mexico; ^5^ Departamento de Formación Básica Disciplinaria, ENMyH-IPN, CDMX, Mexico

**Keywords:** *Toxoplasma gondii*, ocular toxoplasmosis, autoantibodies, HSP70, recoverin, hippocalcin, cerebral toxoplasmosis, cross-reactivity

## Abstract

*Toxoplasma gondii* infection can trigger autoreactivity by different mechanisms. In the case of ocular toxoplasmosis, disruption of the blood-retinal barrier may cause exposure of confined retinal antigens such as recoverin. Besides, cross-reactivity can be induced by molecular mimicry of parasite antigens like HSP70, which shares 76% identity with the human ortholog. Autoreactivity can be a determining factor of clinical manifestations in the eye and in the central nervous system. We performed a prospective observational study to determine the presence of autoantibodies against recoverin and HSP70 by indirect ELISA in the serum of 65 patients with ocular, neuro-ophthalmic and congenital cerebral toxoplasmosis. We found systemic autoantibodies against recoverin and HSP70 in 33.8% and 15.6% of individuals, respectively. The presence of autoantibodies in cases of OT may be related to the severity of clinical manifestations, while in cases with CNS involvement they may have a protective role. Unexpectedly, anti-recoverin antibodies were found in patients with cerebral involvement, without ocular toxoplasmosis; therefore, we analyzed and proved cross-reactivity between recoverin and a brain antigen, hippocalcin, so the immunological phenomenon occurring in one immune-privileged organ (e.g. the central nervous system) could affect the environment of another (egg. the eye).

## Introduction

Autoreactivity is defined as the ability of the immune system components to specifically recognize self-antigens, while autoimmunity is considered a pathological process in which there is tissue damage caused by autoreactive cells and antibodies (1). The etiology of autoimmunity is a complex and dynamic phenomenon, in which genetic and environmental factors are involved; among the latter, the most significant is infection (2). In this study we focused on *Toxoplasma gondii*, an obligate intracellular protozoan, which infects around 30% of humans, most of them asymptomatic (3). This parasitosis is termed *acquired* in cases who got infected by ingestion of contaminated food or water with tissue cysts or oocysts, and *congenital* in children infected during gestation through vertical transmission from the mother. The severity of the disease relies on the virulence of the parasite, the ability of the immune system to control it and, specifically for the congenital infection, the moment of pregnancy when transmission occurs (3–10). Specific forms of clinical toxoplasmosis are ocular (OT) which can occur both in the congenital and acquired types, and cerebral, almost exclusively present in the congenital form (CCT) and in immunocompromised patients (4, 5).

Ocular toxoplasmosis is one of the most important clinical presentations of *T. gondii* acquired and congenital infections, with the development of uveitis or retinochoroiditis (5). It has a significant impact on affected individuals with variable consequences, including partial or total vision loss. Frequency of OT varies from 2% in warm places of the world, to 25% in adults in countries like Brazil and Colombia (11). Patients with congenital toxoplasmosis have retinochoroiditis at birth or develop this problem at any time during the following years, in 80% of cases in adolescence (12). The classical lesion is focalized necrotizing retinochoroiditis, which is the active infection and inflammation of certain layers of the retina involving the choroid. The lesion can be isolated or adjacent to a scar from previous injury; there are less common or atypical OT presentations (13, 14).

On the other hand, congenital toxoplasmosis acquired during the first two thirds of gestation, commonly affects the central nervous system, giving rise to hydrocephalus, microcephaly, or cerebral calcifications, among others. It is not rare that these patients are born with neuro-ophthalmic and even disseminated disease (3, 4, 7).

Besides the damage caused by the parasite and the inflammatory response against it, an immune response triggered against autoantigens could be occurring and aggravating the clinical signs. In fact, several works related to OT and autoimmunity have been published, particularly on detection of antibodies or autoreactive lymphocytes using retinal extracts or isolated antigens -like rhodopsin or S antigen- in humans and rodent or lagomorph experimental models (15–23). Nevertheless, there is no consensus about the role of these responses as a cause or exacerbation of the eye disease; neither there is agreement on the mechanism of autoimmunity elicitation, i.e. if there is a host-parasite cross-reactive antigen (“molecular mimicry”) which stimulates the immune response and causes damage in this way, or there is rupture of the blood-retinal barrier (BRB), and then the autoantigens normally confined to the eye are exposed and recognized as “non-self” by the immune system, which directly responds and damages the tissues (24, 25). Finally, we have only been able to find one investigation that addresses the same question about autoimmunity in cerebral toxoplasmosis: Li et al. demonstrated autoantibodies against the NMDA receptor in chronic patients, which related to behavioral changes and neuropathology (26). These antibodies could be elicited after a temporary break of the blood-brain-barrier (BBB) (25, 26).

In the present study we decided to search autoantibodies against a ubiquitous protein, HSP70, to analyze the “molecular mimicry” hypothesis, since the human molecule shares 76% identity with that of *T. gondii*, and antibodies against the host molecule have been found in experimentally infected mice (27, 28). We decided to study cases with OT, but also included patients with CCT and several with involvement of both organs, i.e. with neuro-ophthalmic alterations.

For the barrier-disruption and autoantigen exposition hypothesis of autoreactivity, we searched antibodies against recoverin, a 23-kDa protein which regulates rhodopsin phosphorylation, expressed exclusively in the photoreceptor layer of the retina and with no ortholog in *T. gondii* (29, 30). Due to interesting results obtained with this antigen, we also included assays with a central nervous system (CNS) confined protein, hippocalcin, which has 51% sequence similarity with recoverin (31).

## Materials and Methods

### Patients and Diagnosis of Toxoplasmosis

For the present study, patients aged 10 days to 60 years with cerebral or ocular toxoplasmosis including active retinochoroiditis, retinochoroidal scars and other ocular complications were recruited. The number of cases studied was 65, 33 pediatric and 32 adults. The patients were classified into different clinical groups: patients with acquired (n = 34) and congenital (n = 6) isolated OT, neuro-ophthalmic congenital toxoplasmosis (n = 16) and CCT without involvement of the eye (n = 9). Samples of seropositive individuals to *T. gondii* without clinical manifestations were also tested (n ​​= 11). Seven sera from healthy individuals seronegative to *T. gondii* were used to establish the cut-offs of serological tests against autoantigens. Due to ethical and cost reasons, no specific studies were conducted to rule out CNS involvement in adults with isolated OT, but they did not have symptoms or record suggesting they presented brain infection.

Patients with previous treatment against *T. gondii* that had ended less than six months prior to the day of recruitment, with chronic infectious diseases other than toxoplasmosis, that had endocrine or autoimmune diseases, and patients participating in another protocol, were excluded from the study.

The ophthalmological examination was carried out in the Clinic of Inflammatory Eye Disease at APEC and in the Ophthalmology department at INP. Retinal examination was performed by fundoscopy and visual acuity impairment by the Teller or Snellen tests; it was defined as normal (>20/40), moderate (20-50 to 20/200) and severe (<20/200). Patients with active retinochoroiditis or retinochoroidal scars, who agreed to participate in the study, or their parents agreed their children to participate, were submitted to a questionnaire and a peripheral blood sample was collected for serum separation, to perform laboratory tests at the Experimental Immunology Laboratory, INP. The laboratory workup for acquired and congenital toxoplasmosis diagnosis includes a panel of standardized serological and molecular tests, including indirect ELISA, western blot, and real-time and end point PCR, described elsewhere (32–35). The avidity test was performed on the positive samples to infer the infection phase (32).

### Indirect ELISA for the Detection of Anti-HSP70, Anti-Recoverin and Anti-Hippocalcin

For the detection of IgG autoantibodies, indirect ELISA was standardized *ad hoc*. The plates were coated either with human recombinant HSP70 (ab78433), recoverin (ab48757) or hippocalcin (ab132300) (Abcam, United Kingdom). Antigens were used at 1μg/mL or 2μg/mL in 0.01M carbonate buffer, pH 9.6, and were incubated overnight at 4°C. Three washes were made with PBS-0.1% Tween 20 (PBS-T) and 200 µl of PBS-Tween with 1.0% BSA (T-BSA) was added as a blocking agent, allowing to incubate for 30 minutes. After washing, 100μL of each sample diluted 1:200 in PBS-T were added per well leaving 2h at 37°C; a standardization step for the determination of this dilution was performed testing some of the samples at 1:200, 1:400, 1:800, 1:1600. Subsequently, after further washes as above, 100 µL/well of anti- human IgG conjugated with peroxidase diluted 1:5000 in PBS-T, were incubated for 2 h. Finally, 100 mL/well of the chromogen solution, containing 4 mg O-phenylenediamine and 0.2% H_2_O_2_ dissolved in 5.0 mL of 0.1M citric acid/0.1M sodium citrate, were added and let to develop for up to 30 min in the dark. The reaction was stopped with 50μL/well of sulfuric acid 0.1M. The absorbance values ​​were obtained in an ELISA reader (Turner Biosystems 9300-010) at 490 nm wavelength and captured with the Modulus Microplate Reader program. The reactivity index (RI) of each sample was calculated by dividing the absorbance of each sample by the cut-off point (average of the negative controls plus three standard deviations). A RI greater than or equal to 1.0 was considered positive.

For the intra-plate positive controls, rabbit anti-human HSP70 polyclonal antibody, rabbit anti-human recoverin polyclonal antibody and mouse anti-human hippocalcin polyclonal antibody (ab79852, ab85292 and ab168214, from Abcam, United Kingdom, respectively) were utilized, and developed with the corresponding conjugates.

### Indirect Competitive ELISA Between Recoverin and Hippocalcin

The test was performed based on the protocol of Kohl & Ascoli, 2017 (36). Plate A was coated with recoverin at 1μg/mL and plate B with hippocalcin at 2.0 μg/mL diluted in carbonate buffer; 100 μL of the antigen dilution were added to each well and incubated 2 h at 37°C. Subsequently, three washes were made with PBS-T, and 200 µL of T-BSA were added to block nonspecific binding, allowing to incubate for 30 min. In plate C, the wells were blocked with 300 μL of T-BSA only; then the selected sera were added in a 1:200 dilution in T-BSA. Each serum was treated on plate C separately with recoverin (at 0, 0.5, 1 and 2 μg/mL) and with hippocalcin (at 0.1, 2 and 2.5 μg/mL). The sera were allowed to incubate with the antigens for 1.5 h at 37°C. The next step was to transfer 100μL of each serum treated with the different concentrations of hippocalcin and recoverin to plates A and B. To both plates (A= recoverin coated and B= hippocalcin coated) we transferred serum treated with the two antigens to be able to analyze autologous and heterologous inhibition in both solid (adhered) and liquid phase. Samples were incubated for 1.5 h at 37C, proceeding with three washes with PBS-T. One hundred microliters of human anti-IgG peroxidase-conjugate diluted 1:5000 in PBS-T were added and left incubating for 1.5 h. Finally, peroxidase activity was revealed as described above. The percentage of inhibition of each sample was plotted versus competitor antigen concentration.

### Avidity Test for Recoverin and Hippocalcin of Selected Samples

Indirect ELISA was performed, adding 6M urea to estimate the avidity of the anti-recoverin and anti-hippocalcin antibodies. Half of the plate was coated with recoverin at 1μg/mL and the other half with hippocalcin at 2μg/mL in carbonate buffer allowing to incubate overnight at 4 C. Three washes were made with PBS-T, and then 200 µl of T-BSA were added, incubating for 30 minutes. After washing, 100μL of each sample diluted 1:200 in PBS-T in quadruplicate were left incubating for 2h at 37 C. After washing again, 2μL of urea 6M in PBS-T were added to two of the wells of each sample and incubated 30 min. After treatment with urea, five washes were made with PBS-T. The reaction was revealed as described above. The avidity of each sample was calculated by dividing the absorbance of the wells washed with urea, by the absorbance of the untreated wells; a value lower than 0.5 was considered low, 0.5-0.65 intermediate and that greater than 0.65, high (32).

### Bioinformatic Analysis

The identity of the amino acid sequences between human HSP70 (NP_005337.2) and HSP70 of *T. gondii* (EPT28683.1) and that of recoverin (NP_002894.1) with hippocalcin (NP_002134.2) as well as the prediction of B antigenic determinants of the three proteins was performed using Blast-NCBI, T-COFFEE and EMBOSS Matcher servers.

### Statistical Analysis

The comparison of the central measures and RI dispersion between clinical groups and subgroups were determined by Mann-Whitney U and Kruskal-Wallis for two or more groups, respectively. Fisher’s exact test was used for significance in bivariate analyses of nominal variables. The Spearman’s test was used to determine the correlation between the autoantibody reactivity indexes to recoverin and hippocalcin and their respective avidity, as well as the reactivity index to the parasite. A p value < 0.05 was considered significant. The SPSS version 22.0 program was used.

### Ethical and Biosafety Considerations

The projects that gave rise to this work were carried out according to ethical principles of the World Medical Association’s Declaration of Helsinki. They were approved by the Reviewing Board of the Instituto Nacional de Pediatría of the Ministry of Health of Mexico (INP; NIH office registrations: IRB-NIH numbers IRB00008064 and IRB00008065), which includes the Investigation, Ethics and Bio-safety Committees (registration numbers 2011/060 and 2016/034). The APEC also registered the project of OT patients, with number IRB UV-18-01.

## Results

### Description of the Population

Sixty-five patients, 32 males and 33 females were recruited, being 17 and 16 pediatric and 15 and 17 adults, respectively. The mean age was 3.7 y (median 5 months) for children and 37.7 (median 35.5 years) for adults. Four of 33 pediatric patients (12%) had acquired toxoplasmosis, and two out of 32 adults (6%) had congenital toxoplasmosis. As stated before, the adult group was composed by isolated OT cases exclusively; while in the pediatric group there were 24% of isolated OT (n=8), 48% neuro-ophthalmic (n=16) and 27% (n=9) with central nervous system disease, but no involvement of the eye.

The clinical characteristics of the 56 cases with OT are described in **Table 1**; isolated OT was predominant, i.e., 40 out of the 56 cases. Regarding laterality, it was observed that bilateral location was more frequent among patients with congenital OT than in those with acquired disease, although activity of the lesions was distributed similarly between the two groups.

**Table 1:** Clinical characteristics of patients with ocular toxoplasmosis.

Variable	Congenital n=22	Acquired n=34	p
Location			
Isolated OT	6 (27%)	34 (100%)	Not applicable
Neuro-ophthalmic	16 (72%)		
Laterality			
Unilateral	9 (41%)	30 (88%)	<0.001*
Bilateral	13 (59%)	4 (11%)	
Retinochoroiditis			
Active	6 (27%)	7 (20%)	ns
Inactive	14 (63%)	27 (79%)	
Location of uveitis			
Anterior	0	2 (6%)	
Posterior	17 (77%)	13 (38%)	<0.001*
Panuveitis	3 (13%)	19 (55%)	
Visual acuity impairment			
None	5 (22%)	7 (20%)	
Moderate	4 (18%)	16 (47%)	0.06
Severe	13 (59%)	11 (32%)	
Location of retinal lesions			
Peripheral	4 (18%)	7 (20%)	
Macular	9 (41%)	15 (44%)	ns
Peripapillary	2 (9%)	4 (11%)	
Multifocal	3 (13%)	4 (11%)	
No lesions	2 (9%)	4 (11%)	
Number of ocular manifestationså			
None	2 (9%)	9 (26%)	
One -Three	14 (63%)	25 (73%)	0.003*
Four or more	6 (27%)	0	
Ocular manifestations	2.38/2	1.31/1	0.02∞
(Mean/Median)			
Number of retinal lesions	1.75/1	1.8/1	ns
(Mean/Median)			
Avidity IgG anti-*T. gondii*			
Low/Acute	5 (22%)	0	
Intermediate	12 (54%)	5 (15%)	<0.001*
High/Chronic	4 (18%)	26 (76%)	

*Fisher’s exact test< 0.01 when compared to SCI-ConD, ∞ Mann-Whitney-U, ns. non-significant.

Other significant differences between congenital and acquired infection were the location of uveitis and the chronicity of the infection, inferred from the avidity test, which as expected was more acute in congenitally infected individuals. The visual quality impairment was differently distributed as well, with a *p* value close to significance. In general, the results indicate that the disease is more recent and more severe in the congenitally infected cases than in those with acquired infection. The specific ocular manifestations associated with retinochoroidal lesions are shown in **Supplementary Table 1**.

### Frequency of Anti-HSP70 and Anti-Recoverin Autoantibodies

**Figure 1:**
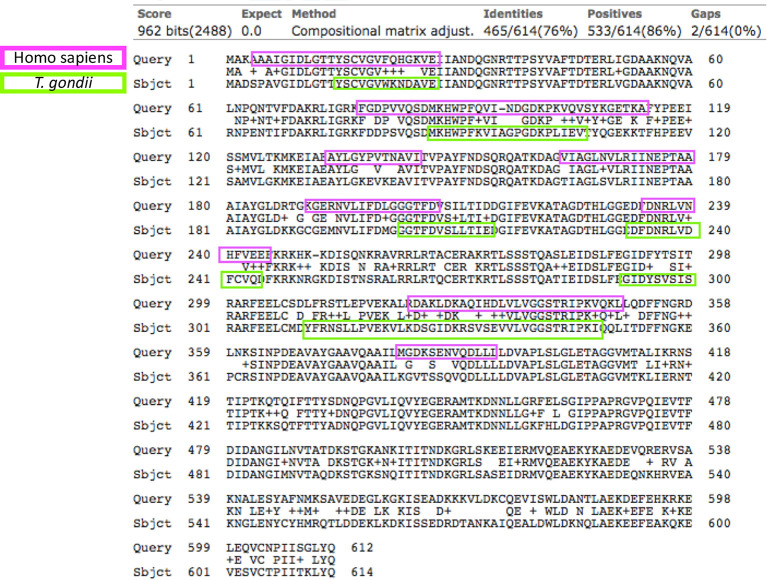
Sequence alignment of the *Homo sapiens* (upper rows) and *T. gondii* HSP70s (lower rows). In the middle lines are the matching amino acids. The identity between the sequences was 76% (yellow circle), in lilac and green colors are the B antigenic determinants predicted for the respective sequences. The arrows indicate the antigenic determinants that both proteins share.

As mentioned before, the sequence identity of human HSP70 and *T. gondii* HSP70 is 76%; the antigenic determinants resulting from the analysis of human HSP70 were eight, five of which were shared between the two species (**Figure 1**). Due to these facts, proving the technical cross-reactivity between human and parasite’s HSP70 was not part of the experimental goals of this study.

**Figure 2:**
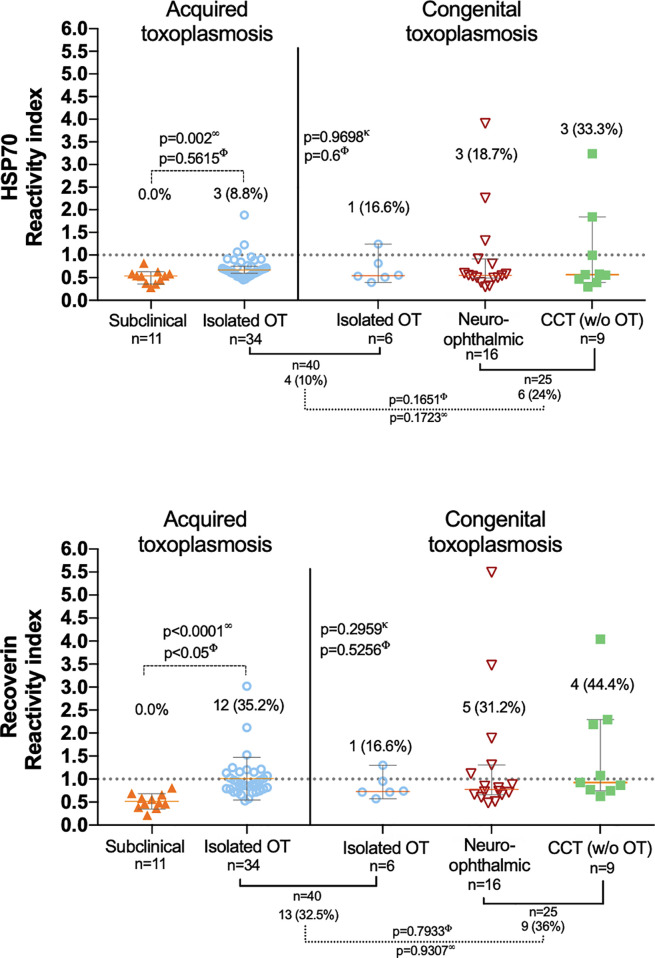
Presence of anti-HSP70 (upper graph) and anti-recoverin (lower graph) autoantibodies in the different clinical groups; the dispersion of the reactivity indices to HSP70 and recoverin is shown, as well as the differences among them (analyzed with ∞ Mann-Whitney U and κ Kruskal-Wallis.) and the proportion of positives RI > 1.0 (analyzed with Φ Fisher’s exact test). The only statistically significant result obtained was the difference between positives in the isolated OT (acquired) and the subclinical group).

The overall proportion of anti-HSP70 and anti-recoverin autoantibodies was 15.6% (10/65) and 33.8% (22/65), respectively, without any subclinical or negative control cases positive for any of them. **Figure 2** shows individual RI and proportion of positives to both HSP70 and recoverin in each clinical group. The differences among congenital infection subgroups, either of RI or positive frequencies against HSP70 or recoverin, were not significant; neither it was the difference between patients with congenital and acquired isolated OT. Only the difference between the subclinical individuals and the acquired OT group attained significance. Nevertheless, some results deserve attention: except for the cases with congenital toxoplasmosis and isolated OT, all groups or subgroups presented a larger proportion of positives against recoverin than against HSP70. Also, among the congenitally infected cases, a higher percent of the cases with CNS involvement presented antibodies as compared to those with isolated OT; unexpectedly, the group with CCT was the one with the highest proportion of autoreactivity, especially to recoverin.

**Figure 3:**
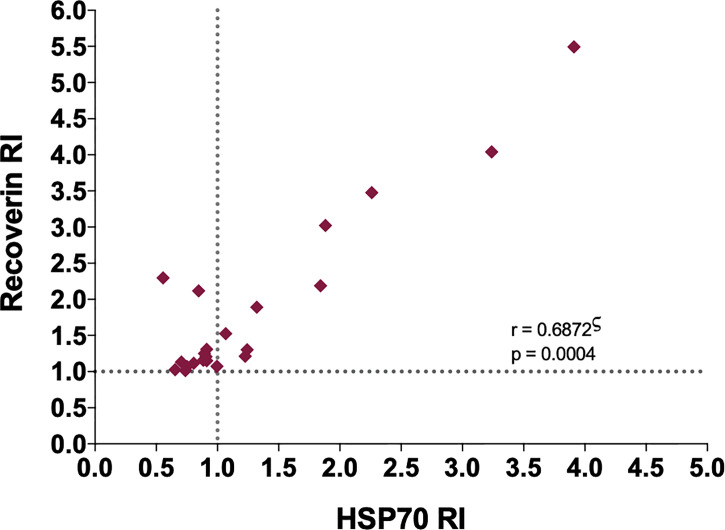
Correlation between the RI to HSP70 and RI to recoverin of the positive samples to one or both antigens. ς Spearman’s correlation.

A significant positive correlation was found between the RI of the samples for HSP70 and recoverin (**Figure 3**), although several cases were negative for HSP70 and positive for recoverin, two of them with high RI values. Bioinformatic analysis showed no identity between the amino acid sequences of the two antigens, and also no antigenic determinants shared between them.

Interestingly, further analysis of the positive samples for anti-HSP70 demonstrated a significant correlation between the RI to *T. gondii* and the RI to HSP70 (r = 0.781, p = 0.01)(**Supplementary Figure 1**). The same analysis was performed plotting RI to recoverin and RI to *T. gondii* which proved no correlation.

### Presence of Autoantibodies and Clinical Characteristics

For patients with OT (n=56), the relation between certain clinical ophthalmological variables and the presence of auto-antibodies was analyzed. Although not significant, some tendencies can be observed; e.g., autoreactivity against HSP70 and recoverin was more frequent among congenital cases with panuveitis, anti-recoverin reaction was prevalent among cases of acquired infection and with anterior uveitis. Likewise, more cases with the acquired form of *T. gondii* infection were positive if the lesions were inactive. However, these differences were not significant. Besides, there was a higher proportion of positives among congenital cases with four or more ocular complications. An apparent relation between autoantibodies against recoverin and impairment of visual acuity was observed for congenital cases and an almost opposite phenomenon occurred in the cases of acquired toxoplasmosis (**Table 2**). In summary, it seems that autoantibodies, especially against recoverin, are associated to more severity in congenitally infected patients and a milder disease in the cases with acquired form of infection.

**Table 2:** Presence of autoantibodies and clinical characteristics of patients with ocular toxoplasmosis.

Variable	Presence of anti-HSP70				Presence of anti-Recoverin			
	Congenital n=22		Acquired n=34		Congenital n=22		Acquired n=34	
Type of uveitis	n	positives (%)	n	positives (%)	n	positives (%)	n	positives (%)
No data	2	1	0	–	2	1	–	–
Anterior	0	–	2	0(0)	0	–	2	1 (50)
Posterior	17	1 (5)	13	2 (15)	17	3 (18)	13	5 (38)
Panuveitis	3	2 (67)	19	1 (5)	3	2 (67)	19	6 (31)
Retinochoroiditis								
No data	2	1	0	–	2	1	0	–
Inactive	14	2 (14)	27	3 (11)	14	3 (21)	27	11 (40)
Active	6	1 (16)	7	0(0)	6	2 (33)	7	1 (14)
Ocular manifestations								
None	2	0(0)	9	1 (11)	2		9	4 (44)
One -Three	14	2 (14)	25	2 (8)	14	3 (21)	25	8 (32)
Four or more	6	2 (33)	0	0(0)	6	3 (50)	0	0(0)
Visual acuity impairment								
None	5	1 (20)	7	1 (14)	5	1 (20)	7	3 (42)
Moderate	4	0(0)	16	2 (12)	4	0(0)	16	8 (50)
Severe	13	3 (23)	11	0(0)	13	5 (38)	11	1 (9)

No significant differences resulted from the analysis of the clinical variables and the presence of auto-antibodies. Fisher, Kruskal-Wallis.

**Figure 4:**
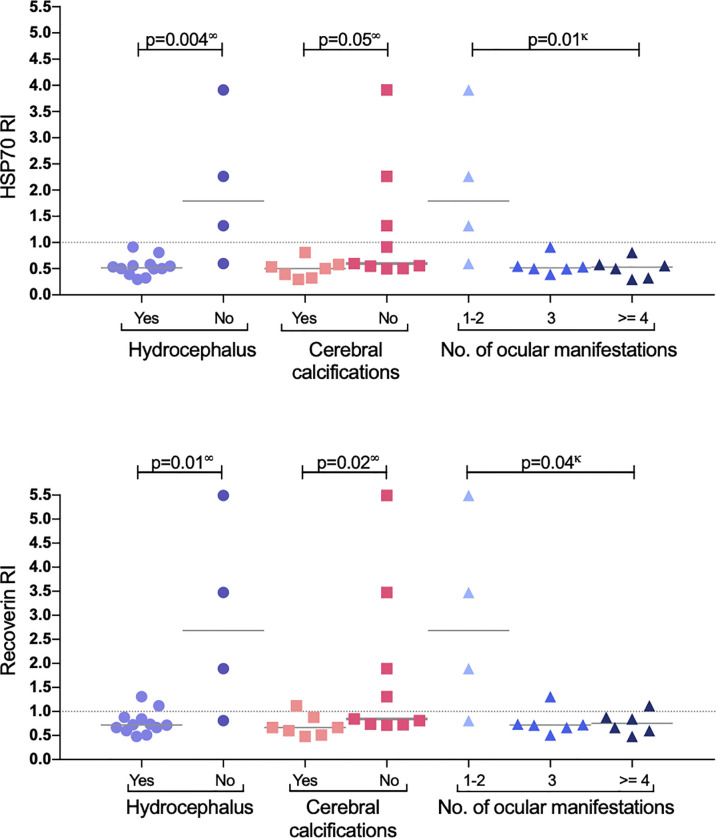
Response to HSP70 (upper graph) and recoverin (lower graph) in the subgroup of neuro-ophthalmic toxoplasmosis. P values using ∞ Mann-Whitney U test, or κ Kruskal-Wallis.

**Figure 5:**
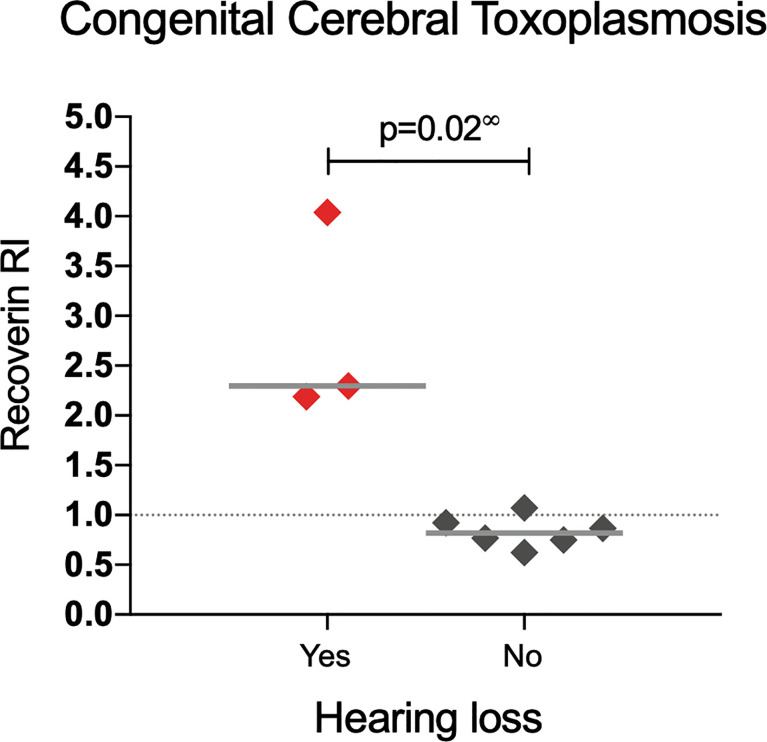
Response to recoverin in patients with congenital cerebral toxoplasmosis without OT. ∞ Mann-Whitney U test.

A similar analysis but for CNS involvement (all congenital cases) is shown in **Table 3**. Interestingly, in patients with involvement of the eye besides the CNS (neuro-ophthalmic) there was an apparent benign effect since autoantibodies were more prevalent among cases with fewer CNS alterations; as shown in **Figure 4**, in the neuro-ophthalmic toxoplasmosis group, significant differences were found in the RI to HSP70 and recoverin between the presence and absence of hydrocephalus and cerebral calcifications. Similarly, the number of ocular manifestations in this group of patients was different with respect to the IR of HSP70 and recoverin, with the category of 1 to 2 complications being the highest proportion of positive individuals compared to those with more ocular manifestations. In patients with CCT without OT, no significant differences were found for these three variables; however, in these individuals, the presence of anti-recoverin autoantibodies was positively associated with the development of hearing loss (**Figure 5**).

**Table 3:** Presence of autoantibodies and clinical characteristics of patients with neuro-ophthalmic toxoplasmosis and CCT without OT.

Clinical manifestation	n		Anti-HSP70	Anti-Recoverin
			Frequency (%)	Frequency (%)
Hydrocephalus				
Neuro-ophthalmic	12		0(0)^∞^	2 (16)^∞^
CCT	4		2 (50)	2 (50)
Cerebral calcifications				
Neuro-ophthalmic	7		0(0)^∞^	1 (14)^∞^
CCT	5		1 (20)	1 (20)
Psychomotor retardation				
Neuro-ophthalmic	16		3 (18)	5 (31)
CCT	8		3 (37)	4 (50)
Hearing loss				
Neuro-ophthalmic	9		1 (11)	3 (3)
CCT	3		2 (66)^∞^	3 (100)^∞^
Seizures				
Neuro-ophthalmic	4			1 (25)
CCT	4		2 (50)	2 (50)
Number of CNS alterations				
Neuro-ophthalmic				
One - Two	4		3 (75)	3 (75)
Three	6			1 (16)
Four or more	6			1 (16)
CCT				
One - Two	5		1 (20)	2 (40)
Three	1			
Four or more	3		2 (66)	2 (66)

∞ Mann-Whitney-U (p < 0.05).

Neuro-opthalmic group n =16.

Congenital Cerebral Toxoplasmosis (CCT) group n = 9.

### Recoverin and Hippocalcin: Identity, Competitive ELISA and Avidity Test

**Figure 6:**
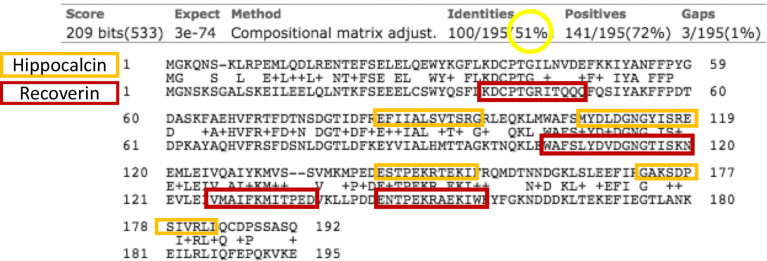
Alignment of hippocalcin (upper row) and recoverin (lower row) sequences. In the middle lines are the matching amino acids. Circulated in yellow is the identity between the sequences, and in the orange and red boxes are the B epitopes predicted in each sequence. The arrows indicate the antigenic determinants that the proteins share.

Due to the results shown above, which demonstrated the presence of anti-recoverin antibodies in patients with CCT without ocular pathology (**Figure 2** green column), we sought other human brain proteins that shared identity and B epitopes with recoverin, since a cross-reaction against a CNS protein by disruption of the BBB was considered as a possible explanation. The protein with the highest homology to recoverin (51%) according to sequence alignments, is hippocalcin. Moreover, these molecules share two B antigenic determinants (**Figure 6**).

**Figure 7:**
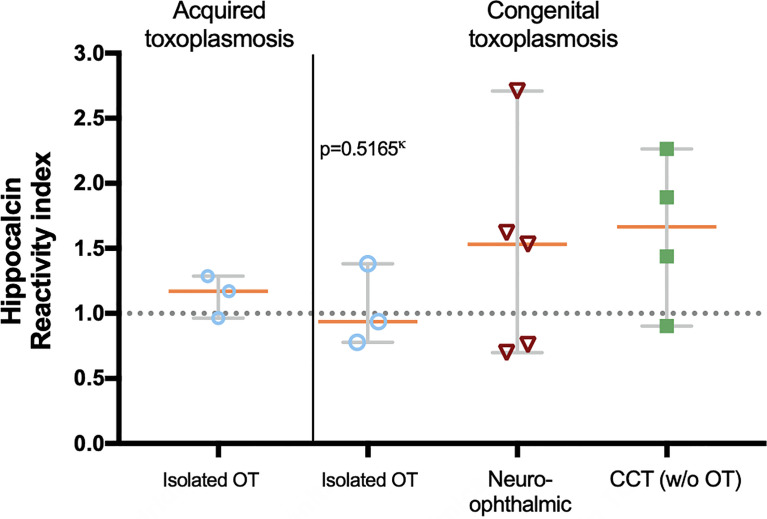
Presence of anti-hippocalcin autoantibodies in the different clinical groups. The graph shows the hippocalcin reactivity of the selected samples and the proportion of positives, i.e. RI ≥ 1.0. Statistics performed with Kruskal-Wallis.

**Figure 8:**
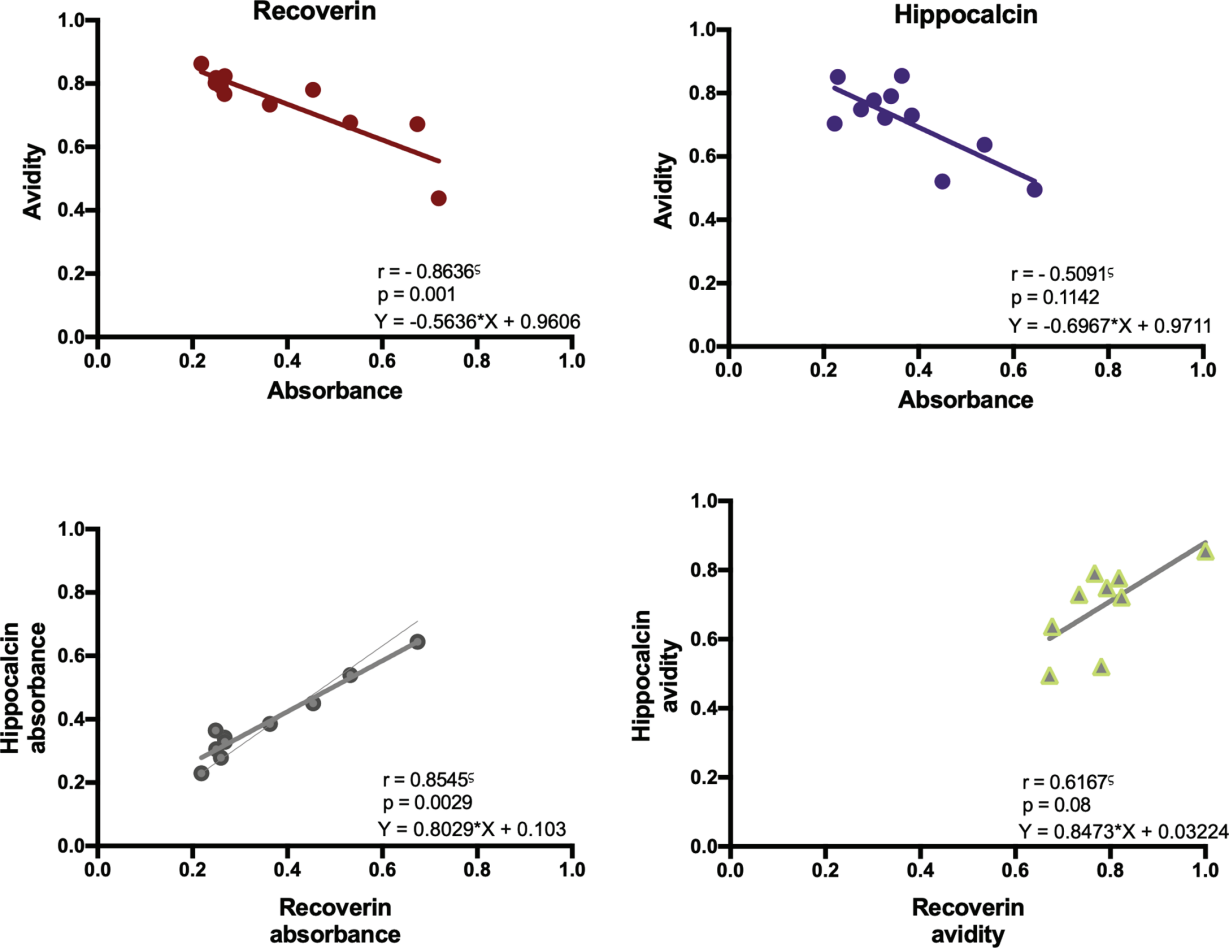
Immunochemical analysis of the anti-recoverin and anti-hippocalcin responses. Correlation between absorbance and avidity for anti-recoverin and anti-hippocalcin. Correlation between absorbance and avidity values to recoverin and hippocalcin. Spearman’s correlation test.

**Figure 9:**
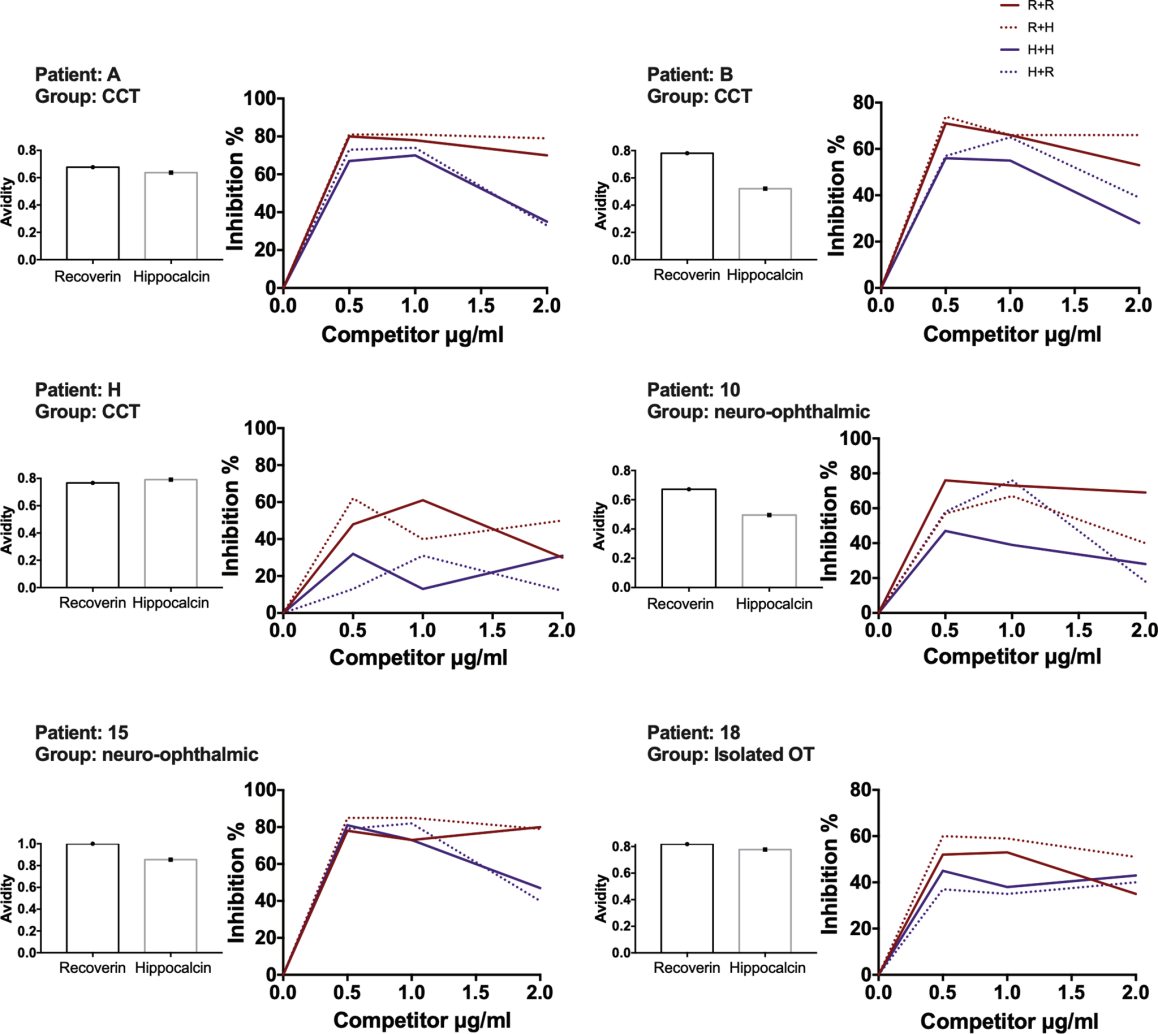
Recoverin-hippocalcin inhibition assay. For each patient the avidity to recoverin and hippocalcin is shown on the left side, the percent inhibition is plotted on the right side according to the added concentration of the competing antigen under four different conditions: – R + R plate coated with recoverin + serum treated with recoverin; - - - R + H plate coated with recoverin + serum treated with hippocalcin; – H + H plate coated with hippocalcin + serum treated with hippocalcin, - - - H + R plate coated with hippocalcin + serum treated with recoverin.

With the above information, 15 samples (twelve positive and three negatives to recoverin) were selected from the different clinical groups and tested for the existence of anti-hippocalcin antibodies (**Figure 7**). Nine of the samples (60%) were positive for hippocalcin, and of the 12 positive samples against recoverin, nine (75%) were also positive for hippocalcin. To get an insight about which auto-antigen was recognized earlier, avidity tests were performed with the double-positive samples. The results are shown in **Figure 8**; and as it can be seen, there is a negative correlation between absorbance and avidity for both antigens, and the correlation between absorbances for both antigens was very high. The avidity of anti-recoverin and anti-hippocalcin antibodies was 0.78 (0.67-0.88) and 0.71 (0.63-0.79), respectively. When plotting the avidity to recoverin against the avidity to hippocalcin (**Figure 8**), it can be observed that for most samples, the avidity is very similar also. In an attempt to further analyze the cross-reactivity between these proteins, a competitive inhibition ELISA was performed, using both antigens at different concentrations, six of the nine samples that were positive for hippocalcin and recoverin were randomly selected (**Figure 9**). The experiment was carried out with both antigens in the solid (adhered) as well as in the liquid phase. In general, the same inhibition pattern is observed in five of the six samples, i.e. the addition of the first concentration of the competing antigen reaches up to 80% inhibition, losing inhibitory effect its last concentration. Although heterologous inhibition is similar to the autologous in most, there was one sample with greater inhibition when adding hippocalcin than when adding recoverin. It should be noted that the avidity for recoverin in samples A, B, 10, 15 and 18 is slightly higher than the avidity for hippocalcin. An exemption of the rule is sample H, which shows a different pattern of inhibition and is the only with higher avidity for hippocalcin than for recoverin (**Figure 9**).

## Discussion

The infectious etiology of autoimmune diseases has been documented in various pathologies (1, 2, 37). During *T. gondii* migration through tissues, it can enter privileged sites like the brain by directly invading endothelial cells, where it replicates, breaking this cell layer thereafter and reaching the encephalon (38). They can also be transported by dendritic cells that act as Trojan horses when trespass to the tissues by diapedesis, delivering the parasite in this way (39). These cells can also act as antigen-presenting cells which lead to pathogen peptides exhibition to T lymphocytes by means of the MHC, class I and II, but also self-antigens that are released from the infected tissue; this triggers a T cell response, which in turn stimulates B cells to produce antibodies (37).

For determining autoantibodies in this study, HSP70 was selected due to its high identity with the *T. gondii* ortholog, and the evidence of cross-reaction between host and parasite HSP70 in a mouse model of toxoplasmosis (28). Supporting that point, in the present study the reactivity of anti-*T.gondii* antibodies correlated to the reactivity level of anti-HSP70 antibodies, while this analysis showed no correlation with those against recoverin. The parasite HSP70 is considered an immunodominant molecule (40). When a microorganism invades, it can be subjected to stress by reactive oxygen species, which induce expression of proteins such as HSPs, making them accessible targets for the immune system. HSPs induce adhesion molecules such as ICAM-1 and VCAM on endothelial cells and the secretion of IL-6, IL-1β and TNF-α. Therefore, HSPs are considered important factors in pathogenic processes that involve cellular necrosis and induction of innate and adaptive pro-inflammatory responses (41).

The frequency of anti-HSP70 and anti-recoverin antibodies was higher in patients with congenital infection and specifically in those with some type of CNS manifestation, even in patients with cerebral toxoplasmosis without ocular infection. Apparently, in patients with OT, autoantibodies against recoverin, are associated to more severity in congenitally infected patients and milder disease in cases with acquired infection. In general terms, our study supports the notion that among the factors involved in clinical outcome is the organ affected.

The literature on autoreactivity against retinal components in the context of ocular toxoplasmosis encompasses the response against S-antigen (arrestin), rhodopsin, inter-photoreceptor-binding protein (IRBP) and extracts from human, bovine or primate retina (15–23). Attempts have been made to determine the association between clinical features in OT and the presence of anti-retinal autoantibodies. However, few significant results have been found. Ten-Berge et al. (42), studied the presence of anti-retinal antibodies in individuals with various causes of uveitis and found a significant association in individuals with moderate visual acuity impairment and active uveitis. In a follow-up study of patients with OT, Abrahams et al., 1982 (16), reported changes in the titers of anti-arrestin that correspond to changes in the ocular pathology, showing their decrease when there was clinical improvement. In general, no association between the presence of anti-retinal antibodies and variables such as the type of uveitis, laterality, the presence of vasculitis, or the size and location of retinal lesions has been demonstrated, which coincides with the present work.

The literature on autoantibodies against CNS proteins in toxoplasmosis is very scarce, mostly studied in the context of psychiatric diseases such as depression, schizophrenia and behavioral changes and particularly for anti-N-methyl-d-aspartate receptor autoantibodies in patients with the chronic infection (26).

The fact that all samples positive for HSP70 were positive for recoverin stands out. There is no bioinformatic nor published evidence about the possibility of cross-reactivity between these two antigens. Sequence and antigenic determinants analysis were performed showing no identity or shared epitopes. Coexistence of antibodies against HSP70 and recoverin was reported in another context: Ohguro et al., 1999 (43) found two bands in a retinal extract in western blot using serum samples from patients with cancer-associated retinopathy, identified as recoverin and HSP70. In another publication (44), the same group experimentally tested the effect of this double reaction in mice injected with antibodies anti-recoverin, anti-HSP70 or both. Retinal damage was demonstrated with anti-recoverin, but not with anti-HSP70 alone, but the severity increased when both antibodies were injected. In our work, the coexistence of these two antibodies seemed to be related to damage in congenital OT cases.

Within the group with neuro-ophthalmic toxoplasmosis an unexpected result was observed: the highest frequency of anti-HSP70 and anti-recoverin antibodies was in patients without hydrocephalus, without cerebral calcifications and with the least number of ocular complications (see **Figure 4**). This negative association may imply a protective effect of autoantibodies within the CNS only in patients that also have the ocular disease, which is difficult to explain, although it might be consistent with a report by Vallochi et al. (20), who analyzed the ability of anti-retinal antibodies to mitigate ocular lesions in OT and showed that patients with milder disease presented higher rate of autoantibodies compared to those who had the severe form. In that sense, different HSP70 epitopes have been identified as MHC-II ligands, and the most studied is the B29 epitope that induces a regulatory T response. *In vitro* studies demonstrated that human HSP60-activated T cells produce Th2 cytokines, while those exposed to bacterial HSP60, responded with a Th1 profile, with INF- γ production. In rheumatoid arthritis, induction of T-cell autoreactivity with HSP60 and HSP70 mitigates the disease in animal models, but when they are inoculated with the microbial ortholog, an enhancing opposite phenomenon occurs (45).

Unexpectedly, we found that anti-recoverin antibodies in patients with congenital cerebral toxoplasmosis without OT seem to have an association with the development of hearing loss, which is a prevalent manifestation in patients with this form of disease, as it can occur in up to 28% of cases without treatment (46). To explain this phenomenon, Salviz et al., 2013 (47), proposed that the inflammation caused by the presence of tachyzoites in the internal auditory canal and in ganglion cells of the inner ear could be damaging the tissues. Nevertheless, no report was found regarding a possible relation of autoimmunity to hearing loss in toxoplasmosis, albeit there is an autoimmune inner ear disease which consists of sensory hearing loss caused by autoantibodies towards components of the cochlea, such as cochlein (48).

The presence of anti-recoverin antibodies in individuals with congenital cerebral toxoplasmosis without ocular pathology may suggest that recoverin epitopes may be shared with another CNS endogenous antigen, resulting in cross-reactivity between the two. The identity of sequences and shared antigenic determinants led us to hippocalcin, identified and cloned by Kobayashi et al. in 1992, which is expressed exclusively in the hippocampus, cerebellum and cortex (49). Importantly the reactivity to these antigens strongly correlated, which may indicate that the antibodies identified in those samples recognize the same epitopes on the two proteins. A higher avidity might indicate greater affinity for the epitope, in that sense, having high avidity towards recoverin may mean that the epitope of the latter is recognized with greater affinity than that of the hippocalcin due to differences in size and conformation. The avidity results suggested that the humoral response to recoverin happened before that to hippocalcin, but they are not conclusive, since the inhibition tests demonstrated a strong cross-reaction between them. Besides, the results may suggest different theories; the presence of both anti-recoverin and anti-hippocalcin antibodies, presence of anti-recoverin with cross-reaction to hippocalcin or anti-hippocalcin with cross-reaction to recoverin; the latter may explain the inhibitory pattern of a sample with higher avidity for hippocalcin and increased inhibition achieved when adding hippocalcin; this patient had no eye involvement.

The line between natural or “protective” autoreactivity and autoimmunity is still unclear. On one hand, autoantibodies are involved in the pathogenesis of numerous autoimmune diseases and cause tissue damage by complement activation, immune complexes, and often their presence involves activation of cellular immunity (50, 51). On the other hand, the protective effect of low-avidity IgM natural autoantibodies is known, which are responsible for inhibiting innate immunity towards self-components by detecting apoptotic cells and promoting phagocytosis of cellular debris (52). Some IgG autoantibodies have also been linked to protection through immunomodulation; examples are antibodies against pro-inflammatory cytokines.

Autoreactivity alone may not be enough to cause disease without some inflammatory or infectious context. This was demonstrated by Voigt et al. in 2017 (53) in a model of transgenic mice expressing an exogenous protein under a specific retinal promoter. The presence of autoreactive T cells against this protein was not sufficient to trigger uveoretinitis; but when they were infected with CMV, which expressed an antigenic epitope of that protein, uveitis and increased lymphocyte proliferation developed.

**Figure 10:**
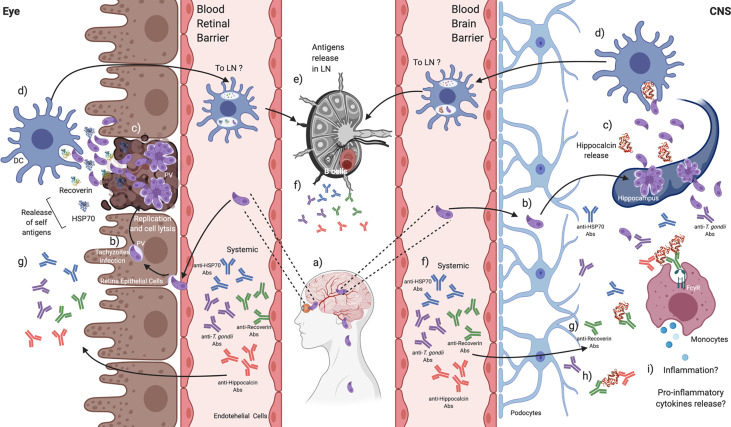
*T. gondii* induces the production of autoantibodies that might be related to disease severity in CNS or the eye. a) *T. gondii *tachyzoites can migrate throughout the blood to reach the CNS and the retina, b) infecting local cells. c) Tachyzoites replication and exit promote lysis, and endogen antigens are released from damaged tissues. d) Tissue-resident DCs could recognize and engulf *T. gondii* tachyzoites and endogen antigens. e) DCs activation can promote its mobilization to the closest lymphatic node (LN) to present antigens, f) probably activating B cells to produce anti-*T. gondii* and anti-endogen antigens antibodies that can be detected systemically. g) These antibodies could reach the retina and the CNS. h) Autoantibodies against recoverin show cross-reactivity against other self-antigens such as hippocalcin, i) which in CNS might be related to increasing a pro-inflammatory environment beneficial against the parasite. Created with BioRender.com.

In summary, this work demonstrated the presence of humoral autoreactivity against a specific retinal antigen, a ubiquitous antigen with cross-reaction with the parasite and a specific CNS antigen, in patients with congenital and acquired toxoplasmosis. Besides, cross-reactivity between a retinal and a CNS protein was identified. It is important to highlight that the presence of autoantibodies was not found exclusively in OT since humoral autoreactivity is also involved in cerebral toxoplasmosis infection, and that these self-reacting macromolecules may be friends or foes, depending on the time and site of infection. Finally, our results also suggest that the autoreactivity induced by an antigen confined in one privileged organ, like the eye, might affect the immune events within other, like the brain (**Figure 10**).

## Conclusions

Autoreactivity against HSP70 and recoverin was found in 15.6% and 33.8% of individuals with ocular, cerebral or neuro-ophthalmic toxoplasmosis, either congenital or acquired.

The severity of clinical manifestations tends to be related to the presence of anti-HSP70 and anti-recoverin autoantibodies in cases of ocular toxoplasmosis, while in patients with CNS involvement, they may have a protective role.

Our data support the existence of cross-reactivity between recoverin and hippocalcin, which opens the possibility that the condition of one organ affects the other in both directions.

## Limitations of the Study

This was a cross-sectional study, which prevents supporting the causal relationship between autoantibodies and the clinical phenotype.

Also, the involvement of the CNS in patients with acquired isolated OT could not be objectively discarded.

## Data Availability Statement

The raw data supporting the conclusions of this article will be made available by the authors, without undue reservation.

## Ethics Statement

The projects that gave rise to this work were carried out according to ethical principles of the World Medical Association’s Declaration of Helsinki. They were approved by the Reviewing Board of the Instituto Nacional de Pediatría of the Ministry of Health of Mexico (INP; NIH office registrations: IRB-NIH numbers IRB00008064 and IRB00008065), which includes the Investigation, Ethics and Bio-safety Committees (registration numbers 2011/060 and 2016/034). The APEC also registered the project of OT patients, with number IRB UV-18-01.

## Author Contributions

MG-M, AI and DC designed the project from which this publication emerged, analyzed the data and were responsible of manuscript drafts and reviews. Besides, MG-M performed all experiments, analyzed the data, and wrote the first draft of the article. FG-C provided scientific and technical advice, helped to write the manuscript and designed the explanatory biological draw. CC-P and DC are the responsible persons of projects 2011/060 and 2016/034 where the present protocol was nested, were responsible for elaboration of the clinical aspects and variables of the projects, as well as for the recruitment of patients. LEC-d-R, participated in the elaboration of the clinical part of project 2016/034 and registered it at APEC; LEC-d-R, RC-K, MS-H, and EO-V recruited the patients and filled all clinical variables of the patients with acquired OT. All authors critically revised the manuscript and contributed to the article and approved the submitted version.

## Funding

This work was partially financed by Centro de Investigación en Ciencias de la Salud (CICSA) (project 201716), FCS, Universidad Anáhuac and Instituto Nacional de Pediatría (project 2016/034), Mexico.

## Conflict of Interest

The authors declare that the research was conducted in the absence of any commercial or financial relationships that could be construed as a potential conflict of interest.

## Supplementary Material

Supplementary Figure 1 Correlation between the RI to HSP70 and RI to *T. gondii* of the positive samples to HSP70. Spearman’s correlation.
