# Supplementary figures and images for: Autoantibodies Against Ubiquitous and Confined Antigens in Patients With Ocular, Neuro-Ophthalmic and Congenital Cerebral Toxoplasmosis
Source: Front Immunol. 2021 May 12;12:606963. doi: 10.3389/fimmu.2021.606963 (PMC8149787; doi:10.3389/fimmu.2021.606963)

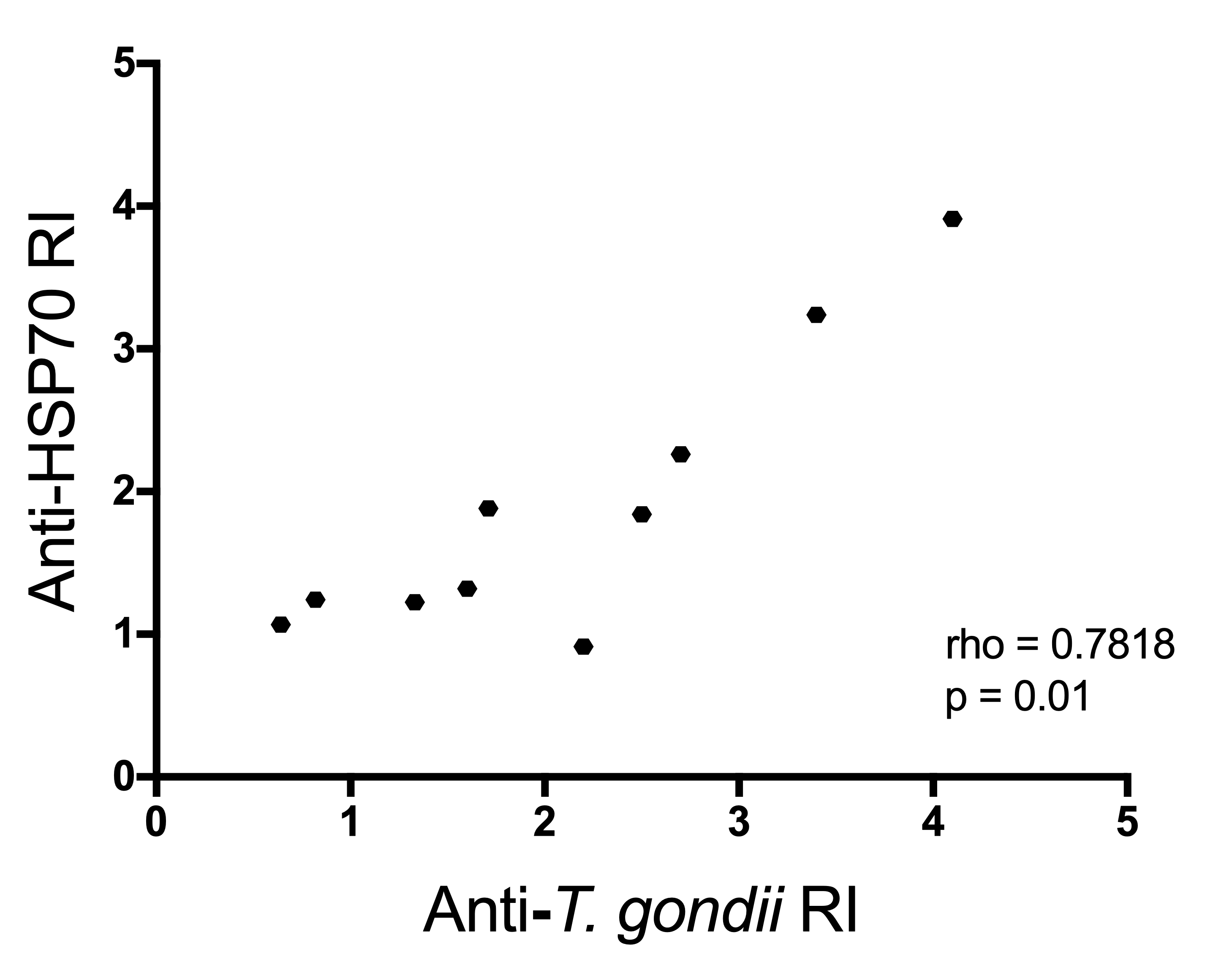

Supplement: Supplementary Figure 1 — Correlation between the RI to HSP70 and RI to T. gondii of the positive samples to HSP70. Spearman’s correlation. [file Image_1.tiff]
